# Attapulgite Structure Reset to Accelerate the Crystal Transformation of Isotactic Polybutene

**DOI:** 10.3390/polym14183820

**Published:** 2022-09-13

**Authors:** Shuang-Dan Mao, Mi Zhang, Fu-Hua Lin, Xiang-Yang Li, Yu-Ying Zhao, Yan-Li Zhang, Yi-Fan Gao, Jun Luo, Xin-De Chen, Bo Wang

**Affiliations:** 1 School of Chemical Engineering and Technology, Taiyuan University of Science and Technology, Taiyuan 030024, China; 2 Shanxi Province Institute of Chemical Industry Co., Ltd., Jinzhong 030621, China; 3 Shanxi Advance Technology Low Carbon Industry Research Institute Co. Ltd., Taiyuan 030021, China; 4 Key Laboratory of Renewable Energy, Chinese Academy of Sciences, Guangzhou 510640, China; 5 Guangzhou Fibre Product Testing and Research Institute, Guangzhou 510220, China

**Keywords:** isotactic polybutene, attapulgite, structure reset, form II to form I, crystal transformation

## Abstract

Isotactic polybutene (iPB) has a wide application in the water pipe field. However, the most valuable form I, needs 7 days to complete the transformation. In this study, the attapulgite (ATP), which produces lattice matching of the iPB form I, was selected to prepare an iPB/ATP composite. The Fischer–Tropsch wax (FTW) was grafted with maleic anhydride to obtain MAFT, and the ATP structure was reset by reactions with MAFT to the prepared FATP, which improved the interface compatibility of the ATP and iPB. The Fourier transform infrared spectroscopy (FT-IR) and the water contact angle test confirmed the successful synthesis of FATP. X-ray diffraction (XRD) verified that the graft of MAFT did not affect the crystal structure of ATP. The iPB + 5% FATP had the maximum flexural strength, which was 12.45 Mpa, and the flexural strength of the iPB + 5% FATP annealing for 1 day was much higher than others. Scanning electron microscope (SEM) photographs verified that FATP and iPB had good interface compatibility. The crystal transformation behavior indicated that the iPB + 5% FATP had the fastest crystal transformation rate, which proved that the reset structure, ATP, greatly accelerated the crystal transformation of iPB. This was a detailed study on the effect of lattice matching, interfacial compatibility and internal lubrication of the reset structure, ATP, in the nucleation and growth stages of iPB form I. The result was verified by XRD, differential scanning calorimetry (DSC), Avrami kinetics and polarizing microscope (POM) analysis.

## 1. Introduction

**Figure 1:**
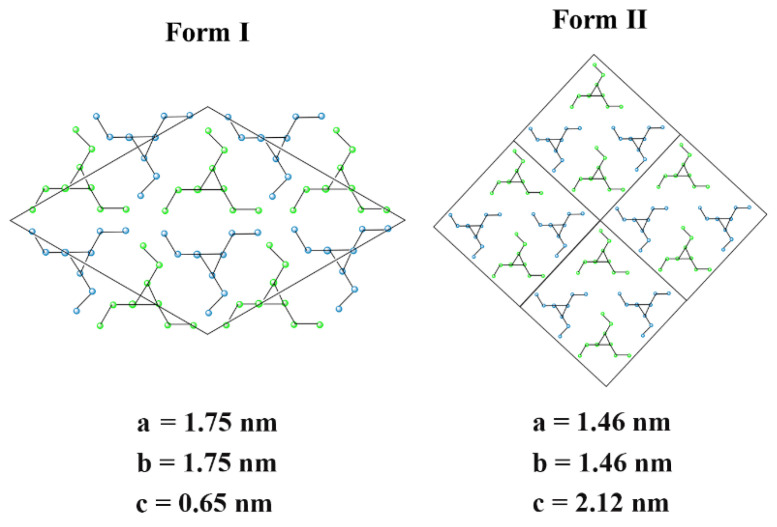
The lattice parameters of iPB form I and form II.

Isotactic polybutene (iPB) is a polycrystalline polymer that has advantages of high creep resistance, excellent elastic recovery and low energy consumption, which has been used in the field of water pipes [1]. The four major crystal forms have been reported for iPB so far, of which the most common crystal forms are form II and form I, and the lattice parameters of form I and form II are shown in Figure 1 [2]. After melting processing, the metastable form II of the iPB is first formed. Spontaneous and irreversible solid–solid crystal transformation slowly takes place during the storage process at room temperature, and the stable form I of the iPB is obtained. The mechanical properties of iPB are greatly improved during the crystal transformation process [3,4]. However, due to the different crystal structure of form II and form I, the process of crystal transformation brings volume shrinkage of products and produce of uneven internal stress, and it takes 7 days to complete the crystal transformation, which greatly increases the difficulty and cost of the iPB processing and production [5]. Therefore, the rapid crystal transformation has great significance for the production and utilization of iPB.

Recently, many methods have been reported to effectively accelerate the transformation from form II to form I, such as controlling melt temperature [6], thermal stress [7], high pressure [8], pressurized CO_2_ [9], copolymerization with random olefin [10], blending with bio-fiber [4,11] and blending with inorganic materials [12].

Blending with inorganic particles can change the crystallization behavior of the polymers due to the effects of heterogeneous nucleation; lattice matching, which has advantages of the simple operation; cheap price; etc. [13]. Previous studies showed that the inorganic particles blending with iPB can increase the nucleation sites of form II and indirectly promote the transformation of iPB crystal, but it cannot directly affect the subsequent transformation process to form I [14,15,16]. The iPB crystallization process is rapid cooling crystallization of form II in the melt, followed by the spontaneous nucleation of form I in the form II phase. Therefore, the crystal transformation rate of iPB is mainly determined by the rate of spontaneous nucleation of form I in the form II phase [17], which indicates that inorganic particles that only nucleate on form II cannot directly accelerate the crystal transformation of iPB. Therefore, the key to accelerate the crystal transformation of iPB is to find a kind of inorganic particle that can induce the nucleation of form I.

The current generally accepted mechanism to promote polymer crystallization is the lattice match, which means that inorganic particles and polymer exist in the structure of one-dimensional or two-dimensional matching when the mismatch rate of geometrical dimensions between inorganic particles and polymer structures is less than 15% [18]. The effect of the lattice match can produce a polymer in the filler surface in strict accordance with one or more structures to determine the crystallographic direction of growth, which prompted polymer crystallization orientation to obtain the target crystal [19]. Therefore, it is of great significance to accelerate the crystal transformation of iPB by blending with inorganic particles, which have lattice matching effects with iPB, and to explore the influence of the crystallographic relationship on the crystal transformation of iPB.

Moreover, the previous research has proved that nucleation and growth temperature have a significant influence on the crystal transformation of iPB [20]. It was found that a few minutes of pre-annealing at −20 °C significantly reduced the time taken to complete the crystal transformation [21]. The reason for this phenomenon is that low temperature annealing is conducive to the generation of crystal nuclei of form I in the crystal of form II. Di Lorenzo et al. [22]. found that form II annealed for 1 h at different temperatures; the transformation at −20 °C can produce the most of form I, while after annealing for 24 h, the transformation at 20 °C yielded the most of form I. Therefore, the relationship between the lattice matching effect and the effect of crystallization temperature regulation on the crystal transformation of iPB still needs to be further clarified.

Attapulgite (ATP) is a kind of clay mineral with a layered chain transition structure [23]. The ATP grows into a nanorod-like crystal form due to the restriction of crystal structure, which has good filling performance and thermal stability [24,25,26]. Above all, the lattice parameter, b = 1.79 nm of ATP, and the lattice parameter, b = 1.75 nm of iPB form I, have the characteristics of a mismatch rate less than 15%, which conforms to the lattice matching theory [27]. Therefore, the ATP can theoretically be used as a filler of iPB to accelerate its crystal transformation through the lattice matching relationship. However, the ATP with strong polarity is incompatible with non-polar iPB, which may further affect the properties of iPB due to the poor interface compatibility. At present, the simplest method to improve the interface compatibility of ATP and iPB is using a coupling agent to treat the surface of ATP [28]. However, in order to ensure the smooth progress of lattice matching, this study chose a more complex way to reset the structure of ATP. The molecular chain segment on the reset ATP can promote the form I molecular chain to enter the lattice smoothly and stack closely after the nucleation of form I through internal lubrication, so as to better accelerate the crystal transformation of iPB.

Fischer–Tropsch Wax (FTW) is a kind of methylene polymer, which has advantages of special fine crystal structure, low oil content, low viscosity and high stability, which can be used as internal lubricant in polymers [29]. However, the lack of active groups on the FTW molecular chain hinders the chemical binding with ATP. Therefore, it is necessary to introduce the carboxyl group into the FTW molecular chain through chemical modification to ensure the combination with ATP through a chemical bond, which can improve the compatibility between ATP and iPB, and play the internal lubrication role of FTW, so as to accelerate the crystal transformation of iPB.

In this study, maleic anhydride was grafted onto the FTW molecular chain to obtain MAFT and the ATP structure reset by reaction with MAFT to prepared FATP. The ATP and FATP were blending with iPB to prepare the iPB/ATP composite. Fourier transform infrared spectroscopy (FT-IR), water contact angle and X-ray diffraction were used to determine the structural and property changes of the ATP. The influence of crystallographic relationship, interfacial compatibility and internal lubrication on the crystal transformation of iPB was discussed in detail. The flexural strength of the iPB/ATP composite was also discussed.

## 2. Materials and Methods

### 2.1. Materials

Isotactic polybutene (iPB, GD025, MFI = 30 g/10 min) was supplied by Orient Hongye Chemical Co., Ltd. (Weifang, China). The ATP was purchased from Jiangsu Aotebang Nonmetal Co., Ltd. (Huai’an, China). The FTW was supplied by Lu’an Chemical Group Co., Ltd. (Changzhi, China). The maleic anhydride, dicumyl peroxide, xylene, acetone, para aminobenzene sulfonic acid, n-hexane were purchased from Tianjin Tianli Chemical Reagent Co., Ltd. (Tianjin, China).

### 2.2. Preparation of FATP

The FTW (5 g) and the maleic anhydride (0.6 g) were dispersed into the xylene (100 mL) to obtain reaction system, then raised the temperature of the system to 120 °C. The dicumyl peroxide was dissolved in 50 mL of xylene with a solid–liquid ratio of 1:2500 (*w:v*), which was dropped into the reaction system that ensured the dripping time was 1.5 h. Then, the rude product was obtained by removing the xylene after reacting for 5 h. Subsequently, the rude product was washed to neutrality by acetone and water and then dried at 70 °C to constant weight. The final product was named MAFT. The hydroxyl index of the MAFT is 35 mg KOH/g, which was tested by titrimetric [30].

**Figure 2:**
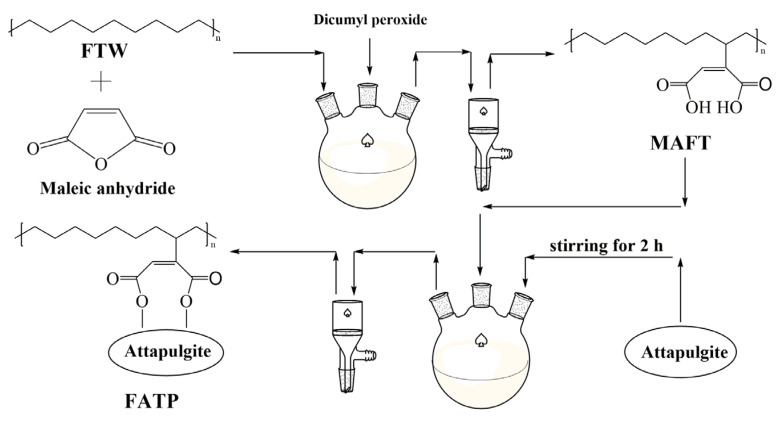
The schematic diagram for the preparation process of FATP.

The ATP (1 g) was dispersed into the xylene (100 mL) with stirring for 2 h to obtain the reaction system and ensured the system temperature was 120 °C. Then, the MAFT (5 g) was added into the system and the temperature of system kept on 120 °C. After the MAFT was dissolved completely, the para aminobenzene sulfonic acid (0.3 g) and the n-hexane (20 mL) were added to the system. After the reaction at 5 h, the rude product was obtained by removing the xylene. Then, the rude product was washed to neutrality by acetone and water and then dried at 70 °C to constant weight. The final product was named FATP. The schematic diagram for the preparation process of FATP was shown in Figure 2.

### 2.3. Characterization of the FATP

The Fourier transform infrared spectroscopy (FT-IR) of the samples were performed on the FT-IR spectrometer (Nicolet iS50, Thermo Scientific Inc., Waltham, MA, USA) using 64 scans per sample.

The ATP and FATP powder were pressed for 5 min at 25 °C under 18 MPa by pressure machine (769YP-10T, Shanghai Xinnuo Instrument Equipment Co., Ltd., Shanghai, China) to obtain ATP and FATP sheet. The hydrophilic of FATP was studied by the water contact angle measuring instrument (DSA25, Kruss, Hamburg, Germany).

The structure of the ATP, MAFT and FATP was tested by X-ray diffraction (XRD) (Rigaku Ultima IV, Rigaku Corporation, Tokyo, Japan) equipped with Cu-Kα radiation (λ = 0.154 nm) and the sample-to-detector distance was 130 mm.

### 2.4. Preparation of iPB/ATP Composite

The formula of the iPB/ATP composite is shown in the Table 1. Each component of the iPB/ATP composite were mixed in a high speed mixer (SHR-500A, Zhangjiagang Ruiteyou Plastic Machinery Co., Ltd., Zhang-Jiagang, China). The evenly mixed samples were fed into the twin-screw extruder (TSH25, Nanjing Chuangbo Machinery Equipment Co., Ltd., Nanjing, China) for extrusion and pelleting to obtain iPB/ATP composite pellets. The dry iPB/ATP composite pellets were molded for standard test specimens by an injection machine (MA1200II, Haitian Plastic Machinery Co., Ltd., Ningbo, China) with an injection pressure of 12 MPa at 150 °C. In addition, the iPB + 5% MAFT composite was prepared by the above method, which was used as a contrast sample.

### 2.5. Characterization of the iPB/ATP Composite

#### 2.5.1. The Flexural Strength of the iPB/ATP Composite

The iPB/ATP composite were annealed at 25 °C for different times (0 day, 1 day, 2 days, 3 days, 5 days, 7 days, 9 days, respectively) after molding in order to study the effects of crystal transformation and interface compatibility on the flexural strength of the composite. The flexural strength of the composite was performed on a universal testing machine (M10, Instron Company, Norwood, MA, USA) according to the GB/T 9341-2008. The crosshead speed was set as 20 mm/min. The five samples for each composite were measured, and the average values were recorded.

#### 2.5.2. The Scanning Electron Microscope (SEM) of the iPB/ATP Composite

The fracture surface of the samples were observed with a SEM (VEGA3, TESCAN, Brno, Czech Republic) at an acceleration voltage of 5 kV.

#### 2.5.3. The Crystal Transformation Behavior of the iPB/ATP Composite

The crystal transformation behavior of the iPB/ATP composite was performed on differential scanning calorimetry (DSC1, Mettler Toledo, Zurich, Switzerland) in a nitrogen atmosphere (50 mL/min). The crystal transformation from form II to form I in different samples, which included the iPB, iPB + 5% ATP, iPB + 5% FATP and iPB + 5% MAFT, were studied by the condition of nucleation at −15 °C and growth at 40 °C. Subsequently, the crystal transformation in nucleation and growth stages was studied, respectively. All samples have eliminated the thermal history from 25–180 °C at 10 °C/min before testing.

The crystal transformation: The samples were cooled to −15 °C at 40 °C/min and kept for 3 min, heated to 40 °C at 10 °C/min and annealed for different times (0 h, 5 h, 16 h, 24 h, 48 h, 72 h, 96 h, 120 h, 144 h), then heated to 180 °C at 10 °C/min, and the melting curve after annealing was analyzed to record the date. The content of form I (*X*_I_) was calculated according to Equation (1) [27].

(1) $$X_{I\;}=\frac{A_{I}/\Delta H_{id,I}}{A_{I}/\Delta H_{id,I}{+A}_{II}/\Delta H_{id,II}}$$

where *A_I_* and *A_II_* were the areas of form I and form II melting peaks, corresponding to the melting enthalpy of two forms. ∆*H_id,I_* and ∆*H_id,II_* were the melting enthalpy of ideal crystals in form I and form II, of which the value was 141 and 62 J/g, respectively [31].

The nucleation stage: The samples were cooled to different nucleation temperatures (0 °C, −10 °C, −15 °C, −20 °C, −30 °C, respectively) at 40 °C/min for 3 min, then heated to 180 °C at 10 °C/min to obtain the melting curve. The influence of nucleation time at −15 °C on the content of form I was also obtained by DSC. The samples were cooled to −15 °C at 40 °C/min for different amounts of time (3 min, 5 min, 10 min, 20 min, 30 min, respectively) and heated to 180 °C at 10 °C/min to obtain the melting curve. The *X*_I_ of the samples was calculated according to Equation (1).

The growth stage: The samples were cooled to −15 °C at 40 °C/min, then heated to 40 °C at 10 °C/min and annealed at 40 °C for different times (0 h, 5 h, 16 h, 24 h) and then heated to 120 °C at a rate of 10 °C/min to melt the form II. Subsequently, they were cooled to 25 °C at 40 °C/min and heated up to 180 °C to obtain the melting curve. The *X*_I_ of the samples was calculated according to Equation (1).

#### 2.5.4. The X-ray Diffraction (XRD) of iPB/ATP Composite

The structure of the iPB/ATP composite was tested by XRD (Rigaku Ultima IV, Rigaku Corporation, Tokyo, Japan), equipped with Cu-Kα radiation (λ = 0.154 nm) and the sample-to-detector distance was 130 mm.

#### 2.5.5. The Polarizing Microscope (POM) of the iPB/ATP Composite

The POM observations of the samples were carried out on a polarized optical microscope (BX51, Olympus Corporation, Tokyo, Japan) and combined with a hot stage (THMS 600, Linkam, UK) to control the temperature. The temperature program was set as follows: the iPB/ATP composite were first heated to 180 °C at 10 °C/min for 3 min, then cooled to 15 °C at 40 °C/min, then annealed at 40 °C for different times (0 h, 5 h, 16 h, 24 h) and then heated to 120 °C at 10 °C/min.

## 3. Result and Discussion

### 3.1. The FT-IR Spectra of the Samples

**Figure 3:**
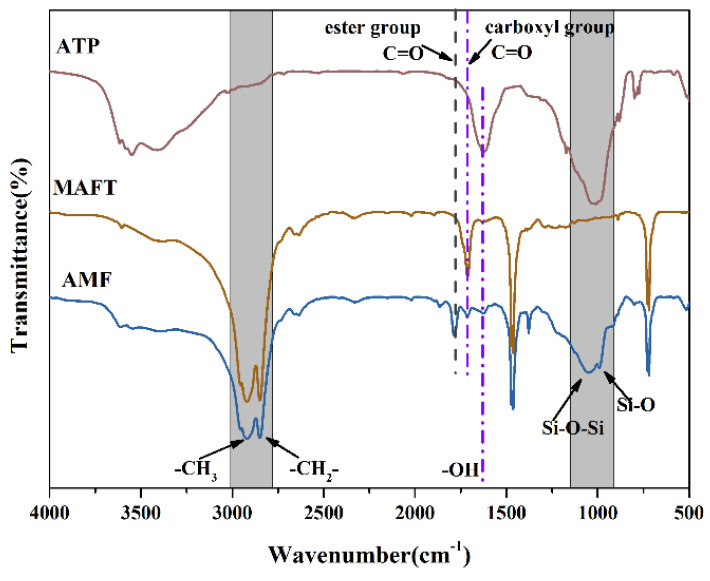
The FT-IR spectra of the samples.

The reset structure of the ATP was confirmed by FT-IR; the FT-IR spectra of the samples are shown in Figure 3. It can be seen from the FT-IR spectra of the ATP that the characteristic absorbance band is between 3392–3616 cm^−1^ representing the -OH of the coordinated water in the tunnels of the ATP, the absorbance peak is 1623 cm^−1^ corresponding to the O-H deformation vibrations of the water and the peaks at 993 cm^−1^ and 1046 cm^−1^ represent the Si-O and the Si-O-Si groups, respectively. In addition, the absorbance peak at 2913 cm^−1^, 2850 cm^−1^, 1459 cm^−1^, 1380 cm^−1^ and 724 cm^−1^ of the FT-IR spectra of the FATP proved the existence of the carbon chains in the FATP. Combining the absorption peak at 1718 cm^−1^, which corresponds to the stretching vibration of the -COOH, the existence of MAFT in the FATP can be demonstrated. Significantly, the new characteristic absorption peak of C=O was located at 1780 cm^−1^. In addition, the absorbance of the O-H in the FATP at 1623 cm^−1^ decreased, compared with the ATP, and the absorbance of -COOH in the FATP at 1718 cm^−1^ deceased, compared with the MAFT [32]. These results proved that esterification occurs between ATP and MAFT, which indicates that the rest of the structure of ATP formed successfully.

### 3.2. The X-ray Diffraction of the Samples

**Figure 4:**
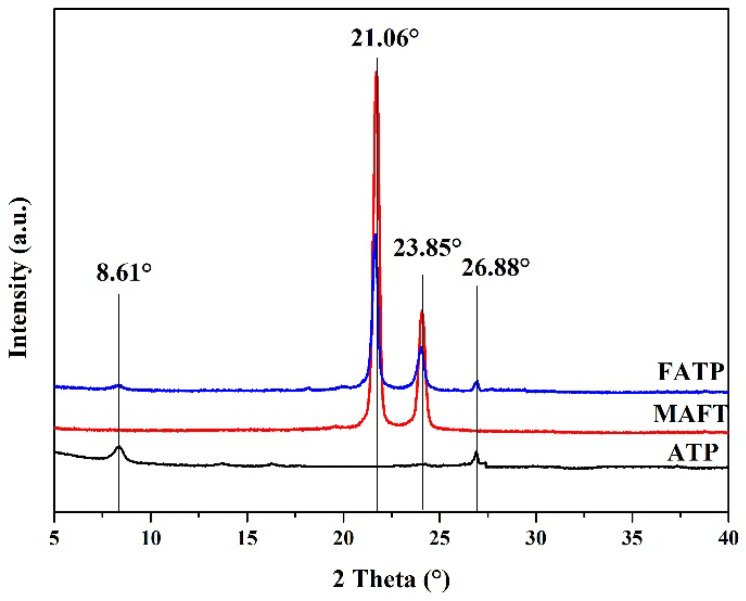
The X-ray diffraction of the samples.

The XRD curves of the samples are shown in Figure 4. It can be seen form the XRD curves of the ATP that the characteristic diffraction peak is at 8.61°, corresponding to the (110) crystal planes of the ATP [33]. The characteristic diffraction peak at 26.88° proved the existence of the SiO_2_ in the ATP [34]. Furthermore, the carbon chain of the MAFT can be verified by the characteristic diffraction peak at 21.06° and 23.85°. For the FATP, the diffraction peaks of the XRD pattern were observed at 8.61°, 26.88°, 21.06° and 23.85°, which were similar to that of ATP and MAFT, respectively, indicating that the MAFT was successfully grafted onto the ATP surface and the graft of the MAFT did not affect the crystal structure of the ATP.

### 3.3. The Hydrophilic Test of the ATP and the FATP

**Figure 5:**
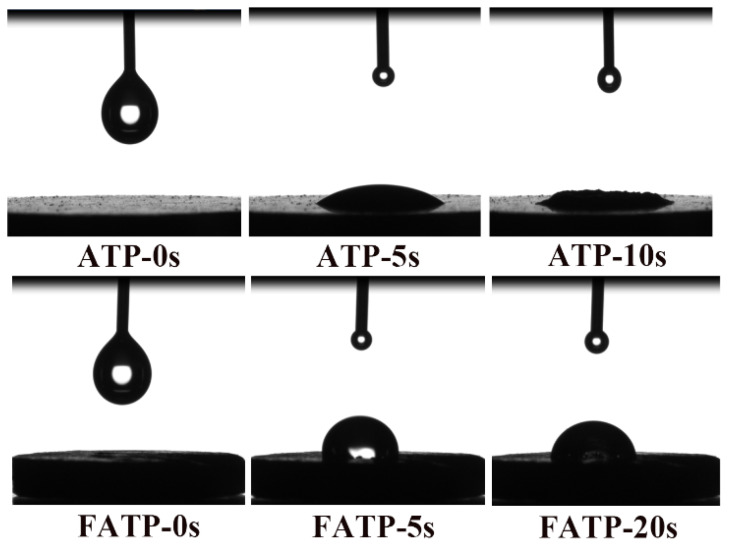
The hydrophilic characterization of the samples.

The water contact angle test was further used to evaluate the hydrophilic characterization of ATP and FATP, and the results are shown in Figure 5. It can be seen from the figure that the water droplet rapidly infiltrated into the ATP when the water droplet contacted the surface of the ATP sheet, and the water droplet was almost entirely gone after 10 s. Meanwhile, the water droplet was hard to infiltrate into the FATP interior by contraries after 20 s. This phenomenon indicated that the reset structure of the ATP can significantly increase the interaction force between the iPB and the ATP, which greatly decreased the hydrophilicity of the ATP, of which the better interfacial compatibility between the ATP and iPB is obtained.

### 3.4. The Flexural Strength of the iPB/ATP Composite

**Figure 6:**
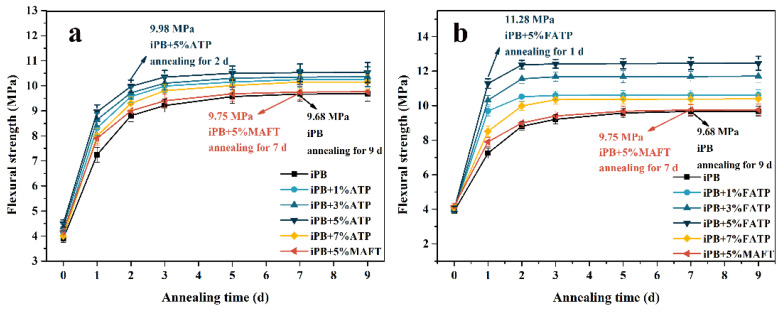
The flexural strength of the iPB/ATP composite. (**a**) The iPB/ATP; (**b**) The iPB/FATP.

The flexural strength result of the iPB/ATP composite with different annealing times is shown in Figure 6. It can be seen from the Figure 6 that the flexural strength of the iPB/ATP composite first increased and then decreased with the increase in ATP or FATP content, and the maximum was 10.52 MPa and 12.45 MPa, respectively, when the ATP or FATP content was 5%. The reason for this result was that the ATP acts as a heterogeneous nucleating agent that can induce the iPB-formed microcrystalline structure, which has stronger rigidity [4]. In addition, the flexural strength of the iPB/FATP samples is always higher than the iPB/ATP samples. This result shows that the structural reset greatly improves the interface compatibility between ATP and iPB, from which the composite can obtain better flexural strength. However, the excess ATP or FATP led to a poor compatibility between iPB and ATP, which caused a decrease in the flexural strength of the composite. In addition, the flexural strength of the composite improved with the extension of the annealing time. The crystal transformation of iPB was completed after 7 days of annealing, and the flexural strength after 9 days of annealing was consistent with the value of the flexural strength after seven days of annealing. The reason for this phenomenon was that the content of form I increased with the extension of annealing time, and form I has the characteristics of high flexural strength [35]. Surprising, the flexural strength of iPB + 5% ATP annealing for 2 days (9.98 MPa) is equal to iPB annealing for 7 days (9.68 MPa). In addition, the flexural strength of iPB + 5% FATP annealing for 1 day (11.28 MPa) was much higher than iPB annealing for 7 days (9.68 MPa), while the iPB + 5% MAFT annealing for 7 days (9.75 MPa) was slightly higher than the iPB. The reason for this result is that the FATP can promote the crystal transformation faster due to the excellent compatibility with the iPB and the lattice matching between the FATP and iPB.

### 3.5. The SEM of the iPB/ATP Composite

**Figure 7:**
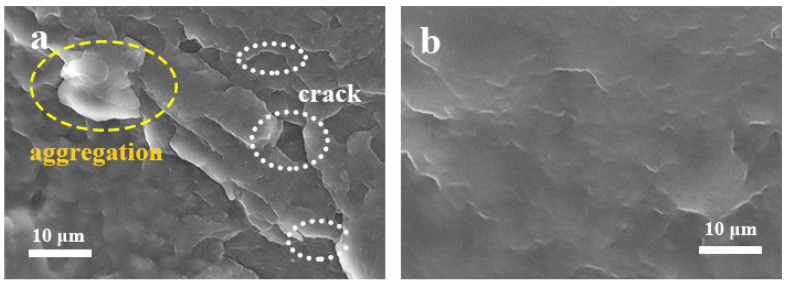
The SEM photograph of the iPB/ATP composite. (**a**) iPB + 5% ATP; (**b**) iPB + 5% FATP.

The fracture surface of the iPB+5% ATP and the iPB + 5% FATP samples were observed by SEM to evaluate the interface compatibility of the composite. As shown in Figure 7, obvious agglomeration and large cracks were observed on the fracture surface of the iPB + 5% ATP, which indicates that the ATP had poor dispersion and weak interaction with the iPB. The iPB + 5% FATP exhibited good dispersion, relatively smooth surface, crack disappearance and uniform particle dispersion. Obviously, the interface compatibility of FATP and iPB was greatly improved. Therefore, the reset structure of the ATP was extremely beneficial in the interfacial compatibility between ATP and with iPB.

### 3.6. The Crystal Transformation Behavior of the iPB/ATP Composite

#### 3.6.1. The Crystal Transformation of the iPB/ATP Composite

**Figure 8:**
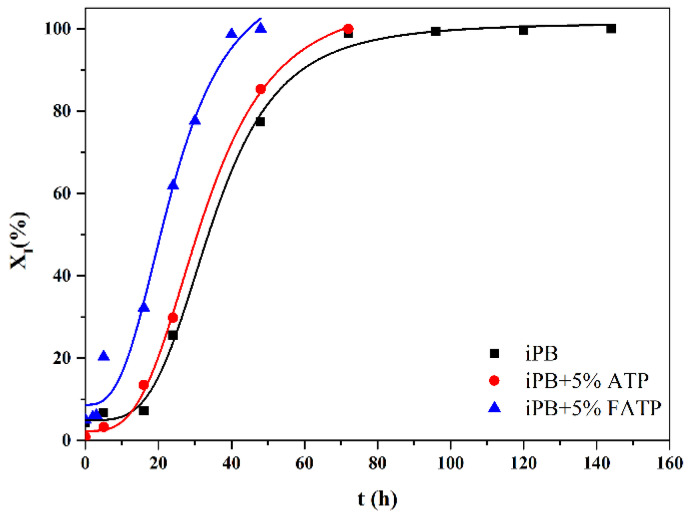
The crystal transformation of the iPB/ATP composite (The solid line was only for guidance and had no mathematical meaning in the figure).

The crystal transformation of different samples after being annealed at 40 °C for different lengths of time is shown in Figure 8. It was clearly seen that the composite spent less time to complete the crystal transformation, compared with the pure iPB (144 h). The iPB + 5% FATP only took 72 h, and the iPB + 5% ATP need 96 h to complete the crystal transformation. The phenomenon suggested that the FATP was more effective at accelerating the crystal transformation of the iPB. As mentioned in the introduction part, the lattice parameters of the ATP partially match those of the form I of iPB, while the FATP has better compatibility with the iPB, compared with the ATP. For the iPB + 5% FATP and iPB + 5% ATP samples with the same crystallization conditions, the only difference was the interface compatibility between the ATP and the iPB, which indicated that the interaction compatibility may also be an important factor to accelerate the crystal transformation of the iPB.

Qin et al. [10] pointed out that the crystallization procedure of form II from melt was fast, but the transformation to form I was slow due to the slow nucleation of form I in the form II phase of the iPB. The lattice matching relationship between the ATP and the form I of iPB reduced the free energy of nucleation (form I), which makes the ATP become a nucleating agent of form I and induces the nucleation of form I rapidly. More nuclei of form I were generated in the iPB due to the improvement of compatibility after the reset structure of the ATP. Therefore, the FATP had a faster rate of crystal transformation. These results indicated that the filler with both lattice matching relationship and good interface compatibility had a more significant promoting effect on the crystal transformation of iPB.

**Figure 9:**
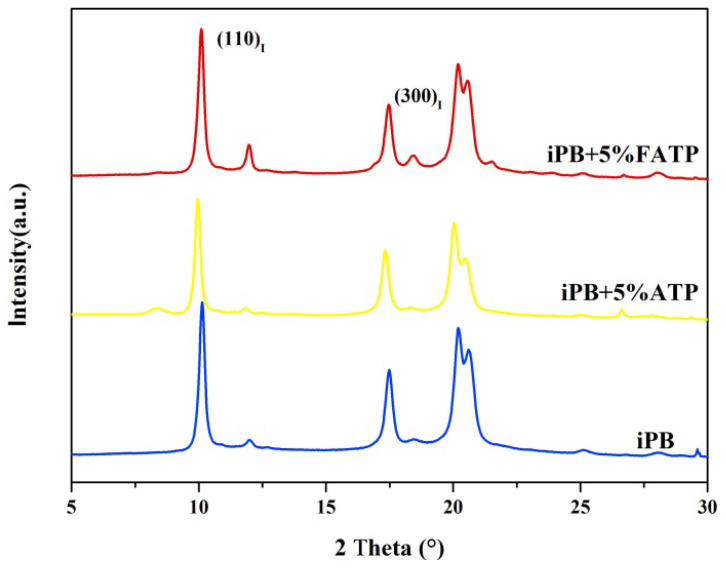
The XRD curves of the iPB/ATP composite.

The XRD curves of the iPB/ATP composite are shown in Figure 9. The intensity of iPB, iPB + 5% ATP and iPB + 5% FATP diffraction peaks at 2θ = 9.9° and 17.3° correspond to the lattice plane (110) and (300) of form I. The previous research showed that when there is no lattice matching relationship between the polymer and the filler, the polymer will not have crystal orientation, and the XRD peak intensity is almost constant [36]. Instead, the XRD peak intensity varies for the filler when the filler and the polymer have a lattice matching relationship. It is clearly seen from the figure that the difference of the diffraction peaks of (110) and (300) crystal planes exists in the samples. In other words, the lattice matching relationship exists between ATP or FATP and iPB. Therefore, the results indicated that the lattice match between ATP or FATP and form I of iPB is the main reason why the ATP has the stronger effect in accelerating the crystal transformation. According to the results in Figure 6, the crystal transformation rate of FATP is faster, which shows that the interface compatibility also has an important influence on the crystal transformation of iPB.

#### 3.6.2. The Crystal Transformation of the iPB/ATP Composite in Nucleation Stage

**Figure 10:**
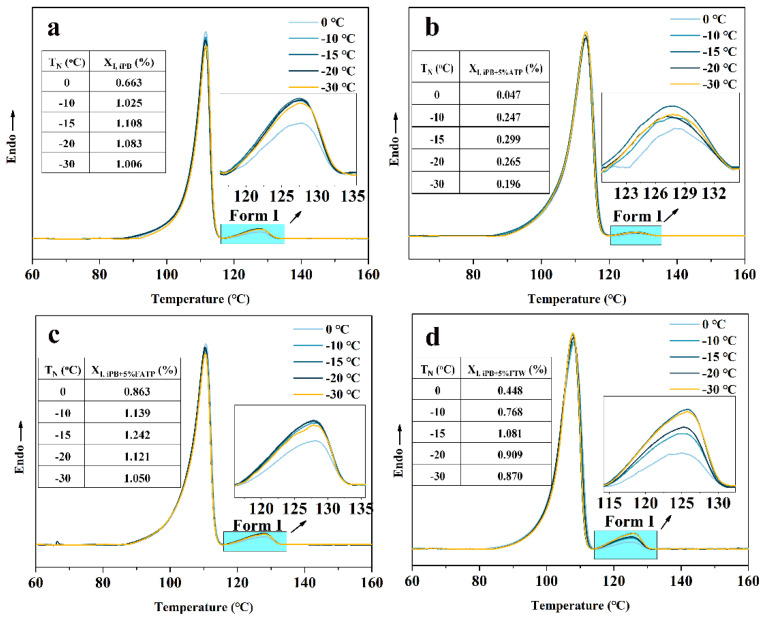
The DSC melting curves of the iPB/ATP composite after being nucleated at different temperatures for 3 min. (**a**) iPB; (**b**) iPB + 5% ATP; (**c**) iPB + 5% FATP; (**d**) iPB +5% MAFT.

The crystal transformation of iPB from form II to form I was affected by the nucleation temperature, and the lower temperature was favorable for the nucleation of form I [17]. The influence of nucleating at different nucleation temperatures (*T_N_*) for 3 min on the content of form I (*X*_I_) was studied, the DSC melting curves of the iPB/ATP composite are shown in Figure 10. The results show that with the increase in *T_N_*, the *X*_I_ of all the samples increased first and then decreased and that most of form I was obtained at −15 °C. This indicated that the low temperature of −15 °C can provide maximum thermodynamic driving force to promote the nucleation of form I. The result is consistent with the results of Qiao’s research [37].

Comparing the *X*_I_ of each sample at −15 °C, the values of *X*_I_ of each sample are in descending order: iPB + 5% ATP < iPB + 5% MAFT < iPB < iPB + 5% FATP. The reason for this phenomenon was that the ATP played a role in rapidly inducing the formation of crystal nuclei of form I due to the effect of lattice matching relationship between ATP and form I. However, the excess ATP had poor compatibility with iPB, which hinders the movement of iPB molecular chains and inhibits the rapid generation of the crystal nucleus of form I. In addition, the *X*_I_ of form I indicated that the MAFT has little effect on inducing the nucleation of form I. However, the FATP containing the ATP and MAFT has the most form I. This is mainly because MAFT provides the composite with good compatibility, which promotes the uniform dispersion of ATP with a lattice matching function in iPB, thus, accelerating the generation of more nuclei of form I. It is not difficult to draw a conclusion that the lattice matching plays a more significant role on the crystal transformation when the filler and polymer have excellent interfacial compatibility.

**Figure 11:**
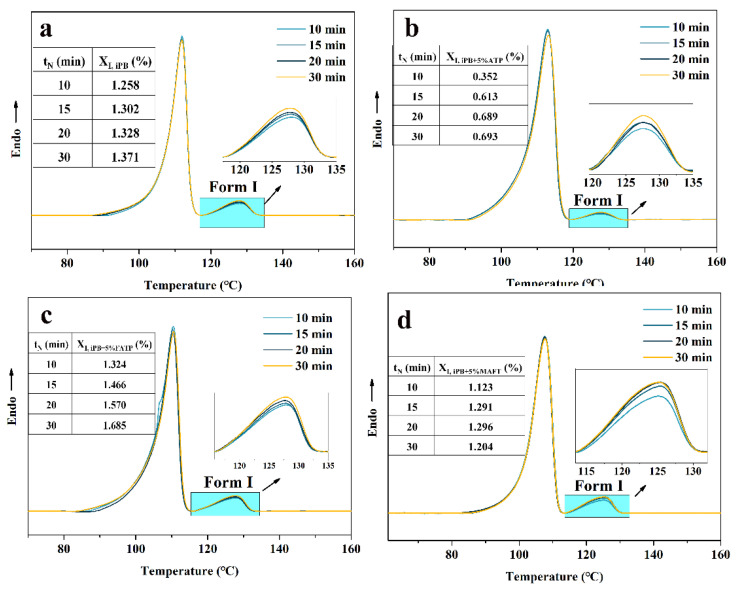
The DSC melting curves of the iPB/ATP composite nucleated at −15 °C for different times. (**a**) iPB; (**b**) iPB +5% ATP; (**c**) iPB + 5% FATP; (**d**) iPB + 5% MAFT.

The nuclear temperature was chosen to be −15 °C and the different nucleation times (*t_N_*) at −15 °C were studied in Figure 11. The results indicated that the *X*_I_ increased with the extension of *t_N_*, which indicated that the increase in *t_N_* had a great influence on the *X*_I_. However, the *X*_I_ of each sample at −15 °C for the same time still followed the rules, in which the order was iPB + 5% ATP < iPB + MAFT < iPB < iPB + 5% FATP. Combined with the conclusion in Figure 10, it can be seen that nucleation of form I required a lower temperature to provide a greater thermodynamic driving force. In addition, the structure reset gives ATP the new function of the interface compatibility and lattice matching, which can promote the crystal transformation of iPB significantly. In the structure reset molecular chains of ATP, the ATP chains can induce the nucleus of form I rapidly through the lattice matching, and the MAFT can improve the interface compatibility of the composite, which can produce more nucleus of form I rapidly. Because of the existence of those two functions, the nucleation rate of form I was greatly accelerated.

#### 3.6.3. The Crystal Transformation of the iPB/ATP Composite in Growth Stage

The nucleation of form I required a lower temperature to provide a greater thermodynamic driving force, at which point the chain diffusion motion required for growth is somewhat suppressed, while crystal growth can proceed rapidly at a higher temperature [38].

**Figure 12:**
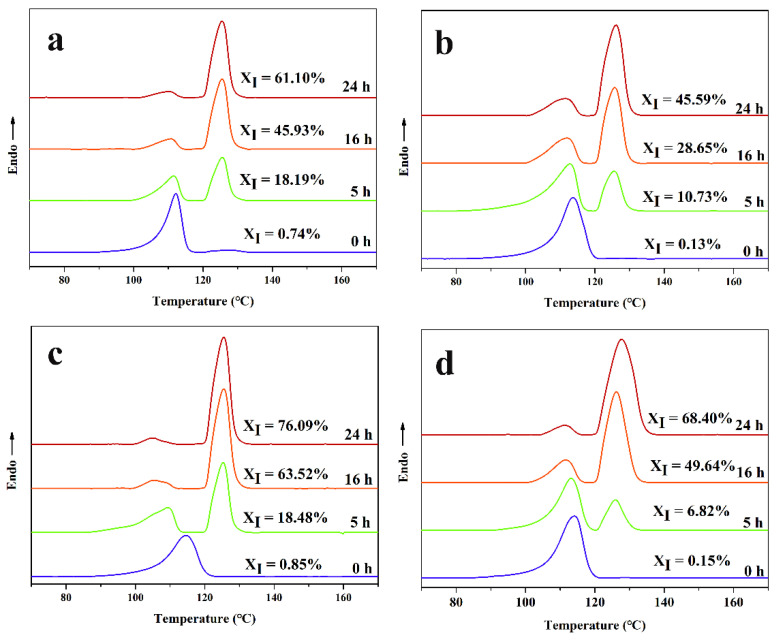
The DSC melting curves of iPB/ATP composite after annealing at 40 °C or different time after thermal treatment at 120 °C (**a**) iPB; (**b**) iPB + 5% ATP; (**c**) iPB + 5% FATP; (**d**) iPB + 5% MAFT.

The thermal treatment temperature of 120 °C was selected to eliminate the form II of the iPB and the growth process of form I was studied. The DSC melting curves of the iPB/ATP composite after thermal treatment at 120 °C are shown in the Figure 12. The results showed that the *X*_I_ of the samples increased gradually with the increase in annealing time. The *X*_I_ of the samples in ascending order was iPB + 5% ATP < iPB < iPB + 5% MAFT < iPB + 5% FATP. The reasons for this phenomenon was that form I remained as an ordered structure and became an effective nucleating agent of form I after eliminating form II at 120 °C, which can realize the rapid crystal transformation. It was worth noting that the iPB + 5% MAFT has more contribution than pure iPB to promoting the crystal transformation of the iPB. The reason for this result was that the MAFT has the function of the internal lubricant, which could accelerate the iPB molecular chain movement to generate more form I in the growth stage. However, this effect is not obvious in the nucleation stage of the composite. Moreover, the iPB + 5% FATP had the best effect of crystal transformation, compared with other samples, which indicated that the internal lubrication effect and good compatibility have brought the molecular chain of ATP through the structural reset, which had a significant effect on promoting the crystal transformation of iPB. This is also the most significant finding of this study.

The crystal transformation kinetics of the composite under growth stage was studied by Avrami method.

The Avrami equation can be expressed as [37]:

(2) $$X_{I}{=1-\exp\;[-kt}^{n}]$$

where *n* is Avrami index and *k* is rate constant. To get *n* and *k* more easily, the equation can be expressed as:

(3) $${\log\;[-\ln\;(1-X}_{I})]=\log k+n\log t$$

**Figure 13:**
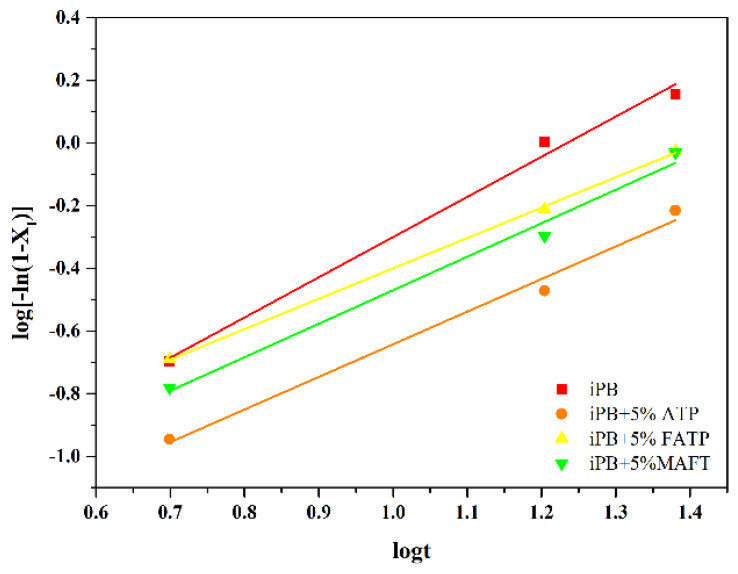
The Avrami kinetics of iPB/ATP composite in the growth stage.

Avrami analysis was performed on the crystal transformation curve in Figure 12 based on the Avrami equation, and the results are shown in Figure 13. Avrami index, *n*, and conversion rate constant, *k*, can be obtained from the slope and the intercept of straight-line fitting, and the results are shown in Table 2. According to the date of Table 2, the value of *n* was close to one for all the samples. The *n* corresponding to one-dimensional growth was one or two, which corresponded to heterogeneous nucleation and homogeneous nucleation, respectively. In this part, the sample retained part of the nucleation of form I, which can be seen as heterogeneous nucleation. The results also showed that all the samples were heterogeneous nucleation and one-dimensional growth mode, especially the iPB sample. The conversion rate constant, *k,* from small to large was iPB + 5% ATP < iPB < iPB + 5% MAFT < iPB + 5% FATP, which further showed that the internal lubrication affect and good compatibility have brought the molecular chain of ATP through structural reset, which had a significant effect on promoting the crystal transformation of iPB.

**Figure 14:**
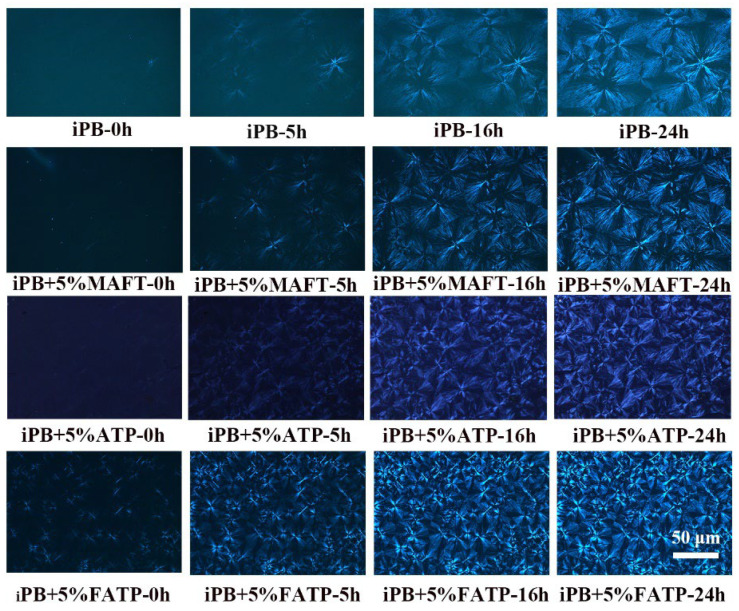
The POM images of iPB/ATP composite.

All the samples were annealed for different times (0 h, 5 h, 16 h, 24 h) at 40 °C and then heated to 120 °C to eliminate the form II of the iPB and the POM images are shown in Figure 14. It can be seen from the POM images of the iPB sample that the crystals in the field of vision are form I of the iPB, while form II of the iPB was completely melted at 120 °C. It was not difficult to find that the addition of the ATP increased the density of the crystal nucleus and decreased the spherulite size. After annealing for 5 h, the spherulite of iPB + 5% FATP were significantly more than those of other samples. After annealing for 16 h, the edge of iPB spherulite darkened and the boundary between crystals was blurred, from which the unmelted crystals was form I. The edge of iPB + 5% MAFT crystals darkened and a clear boundary between crystals could be observed to show a small portion melting, and the iPB + 5% ATP had little of form I, while iPB + 5% MAFT crystal boundary was obvious, and the crystal structure was complete. The form I content of the samples increases from small to large was iPB + 5% ATP < iPB < iPB + 5% MAFT < iPB + 5% FATP, which further verifies the change in *X*_I_ during the growth process. The results show that iPB + 5% FATP had the most form I at 120 °C for the same annealing time. This is also the intuitive evidence that FATP can better promote the crystal transformation of iPB.

In conclusion, the reset structure ATP can introduce the effect of lattice matching, interfacial compatibility and internal lubrication into the ATP molecular chain. The effect of lattice matching and interfacial compatibility play a major role in the nucleation stage of iPB form I, and the effect of lattice matching and internal lubrication play a major role in the growth stage of iPB form I. The reset structure ATP greatly accelerates the crystal transformation of iPB due to the synergy of these effects.

## 4. Conclusions

In this study, the ATP, which has lattice matching of the iPB form I was selected as the filler to study its role in the iPB crystal transformation process. The structure of ATP was reset by graft with MAFT in order to improve the interface compatibility of the ATP and iPB and the iPB/ATP composite were prepared by the melt blending method. The FT-IR confirmed the successful synthesis of FATP, and the water contact angle test showed that the hydrophilicity of FATP was greatly reduced. The XRD verified that the graft of the MAFT did not affect the crystal structure of ATP. The results of flexural strength indicated that the iPB + 5% FATP had the maximum flexural strength, which was 12.45 MPa and the flexural strength of the iPB + 5% FATP annealing for 1 day (11.28 MPa) is much higher than the iPB annealing for 7 days (9.68 MPa), the iPB + 5% ATP for 2 days (9.98 MPa) and the iPB + 5% MAFT annealing for 7 days (9.75 MPa), respectively. The SEM result proved the reset structure of the ATP has been extremely beneficial in the interfacial compatibility between ATP and with iPB. Moreover, the results of crystal transformation behavior of the iPB/ATP composite indicated that the iPB + 5% FATP had the fastest crystal transformation rate and the form II can be completely transformed into form I in only 48 h, while the iPB + 5% ATP took 72 h, but the iPB required 144 h for complete transformation. The XRD confirms the lattice matching relationship exists between ATP or FATP and iPB. The results of the crystal transformation in nucleation and growth stages of iPB indicated that the −15 °C nucleation is favorable for the generation of form I, which is mainly because the thermal stress brought by the cooling process promotes the generation of form I. In the nucleation stage, the *X*_I_ of each sample is in descending order: iPB + 5% ATP < iPB + 5% MAFT < iPB <iPB + 5% FATP. It was indicated that the FATP with both lattice matching relationship and good compatibility had more significant promoting effect on iPB crystal transformation. In the growth stage, The *X*_I_ of the samples in ascending order was iPB + 5% ATP < iPB < iPB + 5% MAFT < iPB + 5% FATP, which was different from the nucleation stage. The reason for this phenomenon is that the ATP chains can induce the nucleus of form I rapidly through lattice matching, and the MAFT chains can improve the interface compatibility of the composite, which can produce more of a nucleus of form I rapidly, and the result was verified by Avrami dynamics. The POM also confirmed this conclusion. In conclusion, the effect of lattice matching and interfacial compatibility play a major role in the nucleation stage of iPB form I, and the effect of lattice matching and internal lubrication play a major role in the growth stage of iPB form I. The reset structure ATP greatly accelerates the crystal transformation of iPB due to the synergy of these effects.

## Figures and Tables

**Table 1:** The formula of the iPB/ATP composite.

Sample	iPB/g	ATP/g	FATP/g	MAFT/g
iPB	1500	0	0	0
iPB + 1% ATP	1500	15	0	0
iPB + 3% ATP	1500	45	0	0
iPB + 5% ATP	1500	75	0	0
iPB + 7% ATP	1500	105	0	0
iPB + 1% FATP	1500	0	15	0
iPB + 3% FATP	1500	0	45	0
iPB + 5% FATP	1500	0	75	0
iPB + 7% FATP	1500	0	105	0
iPB + 5% MAFT	1500	0	0	75

**Table 2:** The parameter of the analysis of Avrami.

	iPB	iPB + 5% MAFT	iPB + 5% ATP	iPB + 5% FATP
*n*	1.282	0.969	1.041	1.068
*k*	0.026	0.029	0.021	0.043

## Data Availability

Not applicable.
